# Salmon Louse Infestation Impairs the Long‐Term Survival of Sea‐Run Brown Trout

**DOI:** 10.1002/ece3.71006

**Published:** 2025-08-12

**Authors:** Knut Wiik Vollset, Bjørnar Skår, Robert J. Lennox, Rosa Maria Serra‐Llinares, Gunnar Bekke Lehmann

**Affiliations:** ^1^ NORCE Norwegian Research Centre Laboratory for Freshwater Ecology and Inland Fisheries Bergen Norway; ^2^ Ocean Tracking Network Dalhousie University Halifax Nova Scotia Canada; ^3^ Institute of Marine Research Tromsø Norway

**Keywords:** aquaculture, brown trout, parasite, salmon louse, sea lice, spill‐back

## Abstract

Anadromous salmonids, including sea‐run brown trout, are exposed to ectoparasitic salmon lice during their sea migrations. The development of intensive aquaculture in coastal areas has promoted louse epidemics by substantially increasing the number of hosts available to the parasite. We employed a mark‐recapture study involving large‐scale traps to capture and PIT‐tag 676 wild sea‐trout during their early marine migration in spring 2020 and 2021. Each trout was examined for lice, tagged with passive integrated transponders (PIT), and monitored for subsequent survival using a PIT antenna system installed at the river Yndesdalsvassdraget. Using a Cormack‐Jolly‐Seber capture recapture model of individual re‐detections the subsequent years, we found a significant negative correlation between lice per gram of fish weight and the survival probability. Increasing lice load from 0 to 1 louse per gram fish reduced the survival probability by approximately 73% in 2020 and 58% in 2021. This is among the first field studies to demonstrate a statistically significant association between individual survival of brown trout and their parasite loads in the wild. Our findings demonstrate the critical need for robust marine spatial planning and lice management in coastal fisheries. Effective control of lice loads is essential to mitigate their deleterious effects on brown trout, ensuring sustainable fish populations and maintaining ecological balance in regions affected by aquaculture.

## Graphical abstract

Salmon louse ( *Lepeophtheirus salmonis* ) infestations significantly impair the survival of sea‐run brown trout ( *Salmo trutta* ), with a 73% reduction in survival probability per louse per gram of fish weight in 2020 and a 58% reduction in 2021. Our findings provide clear evidence of the long‐term ecological impacts of parasite burdens and underscore the critical need for improved parasite management in aquaculture‐affected regions.

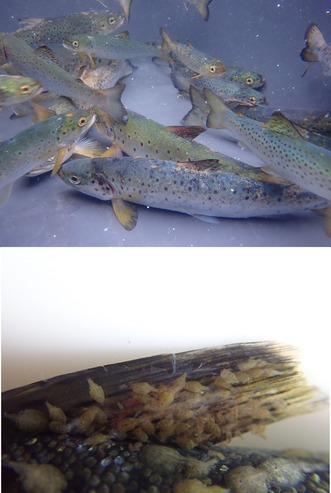

## 1 Introduction

The collateral effects of augmenting parasite production in a marine area due to fish farming is one of the most pressing environmental impacts in salmon farming in the North Atlantic Ocean (Forseth et al. 2017). For wild living sea‐run brown trout ( *Salmo trutta* ) (from here onward only sea trout), the ectoparasitic salmon lice ( *Lepeophtheirus salmonis* ) has been defined as the main anthropogenic cause of poor conditions of populations throughout Norway (Fiske et al. 2024). The life cycle of the salmon louse begins with free‐swimming larvae that hatch from eggs released from egg strings on the mature female. These develop through nauplius and copepodite stages before attaching to a host fish. They then develop through two attached stages (chalimus), after which they become mobile (preadult) until reaching adulthood (adult) and reproducing to start the cycle anew (Hamre et al. 2013). In general, the salmon louse starts to exert a large impact on their hosts as the lice moults from the attached stages (copepodid and chalimus 1 and 2) to the mobile (preadult and adult stages; Wagner et al. 2008). During the larger mobile life stages, the lice can generate substantial wounds on its host as it consumes the mucus, skin, and blood of the fish. One impact of this parasitic interaction is that the fish struggles with osmoregulation as the water/body barrier is broken, dehydrating the animal by osmosis, which will lead to mortality without corrective action (Birkeland and Jakobsen 1997). These dermal wounds often occur on the head and around the anal fin where the salmon lice aggregate, but also on the dorsal fin. Trout that have been subjected to heavy parasitism by these marine crustaceans are often scarred, bloodied, and seeking out freshwater to rebalance their internal osmotic gradient, which reduces their marine living time and affects growth and fitness (Thorstad et al. 2015; Finstad et al. 2021).

For the closest relative of brown trout in the north Atlantic, the Atlantic salmon ( *Salmo salar* ), parasitism by *Lepeophtheirus salmonis* can lead to death either through direct effects or through secondary infections if the abundance of lice is large enough (Finstad et al. 2021). To quantify this mortality effect on wild populations, thresholds of how many lice migrating post‐smolt of wild salmon can tolerate are used to define mortality probability according to equations derived from laboratory trials (Taranger et al. 2015; Ives et al. 2023). These experimentally determined thresholds are a key component of the political fish farming regulation systems that have been developed to assess the environmental impact of salmon lice in Norway (Vollset et al. 2014) and are also emerging in other countries (Moriarty et al. 2023). Applying the same frameworks used for Atlantic salmon to understand the impacts of lice on wild sea trout is challenging, in large part because of the different life history and behaviour of the sea trout (Birnie‐Gauvin et al. 2023). Unlike Atlantic salmon that migrate long distances out to sea, the trout generally stays close to the coast, having prolonged exposure to lice and also to sources of freshwater that can be used to recuperate from the osmotic damages caused by lice exposure (Finstad et al. 2021).

Early research on salmon lice (Birkeland and Jakobsen 1997) demonstrated that sea trout have the ability to return to freshwater as a means of compensating for the osmoregulation failure caused by salmon louse infestations. This adaptive behaviour helps the trout restore their salt balance, offering a crucial survival strategy in response to the stress and damage inflicted by the parasites in marine environments. Freshwater can also remove parasites from the host as the salmon lice is marine stenohaline (Sievers et al. 2019). Therefore, the impact of salmon lice on sea trout is strongly influenced by the trout's access to freshwater habitats and their behaviour, because frequent or timely returns to freshwater can significantly reduce parasite loads and associated physiological stress. Consequently, calculating the effect of salmon lice infestation on survival of affected trout is inherently complex. Studies have shown correlative associations between infestation pressure of lice and return rates of sea trout (Gargan et al. 2003), and potential impact of presence of farms on the population trajectories of sea trout (Butler 2002; Walker et al. 2006). However, such studies are often criticised due to the potential for spurious correlations. In addition, previous studies have not succeeded in estimating individual tolerance levels, which is a necessary factor to provide to some management systems (Vollset et al. 2014). Estimates of the individual impact of salmon lice on survival and premature returns of sea trout, have been made using acoustic telemetry and experimental infestations of trout with controlled doses of lice (Serra‐Llinares et al. 2020). Similar studies have been done on Arctic charr ( *Salvelinus alpinus* ) (Strøm et al. 2022). These studies have found significant effects on trout behaviour, where infested fish seek surface waters and stay on average closer to the river compared to non‐infested fish. However, the low replication due to cost of tags and their relatively short battery life means that any estimates of impact on overall survival is generally not possible across years. Indeed, power analysis of comprehensive field studies using this method has revealed that the power to detect a significant effect on overall survival is low (Serra‐Llinares et al. 2020).

Passive integrated transponders (PIT) offer an alternative method to other tracking technologies that allow the accumulation of long term individual data on the impact of salmon louse on sea trout. PIT tags allow for potential detection across the lifetime of the trout, and in combination with capture recapture models from data across multiple years, this telemetry system makes it possible to calculate the probability of surviving across the lifetime of the animal as a function of parasite load at capture. In this study, our goal was to estimate the long‐term effect on survival of salmon louse levels on sea trout. By tagging fish caught in trap nets in the estuary of the River Yndesdalsvassdraget, and registering on a river antenna and recaptures at the trap the consecutive years, our goal was to use a mark‐recapture estimate of the effect of salmon lice on overall lifetime survival of sea trout. This estimate could be a foundation for an in situ measurement of the tolerable level of salmon louse that is necessary to be able to implement assessment of sea trout in the fish farming management system. We hypothesised that increased salmon louse infestation levels on sea trout will correlate with a significant reduction in overall lifetime survival, with fish experiencing higher parasite loads exhibiting lower recapture rates and reduced longevity compared to those with lower infestation levels.

## 2 Methods

### 2.1 Study Area and Capture Locations

**FIGURE 1:**
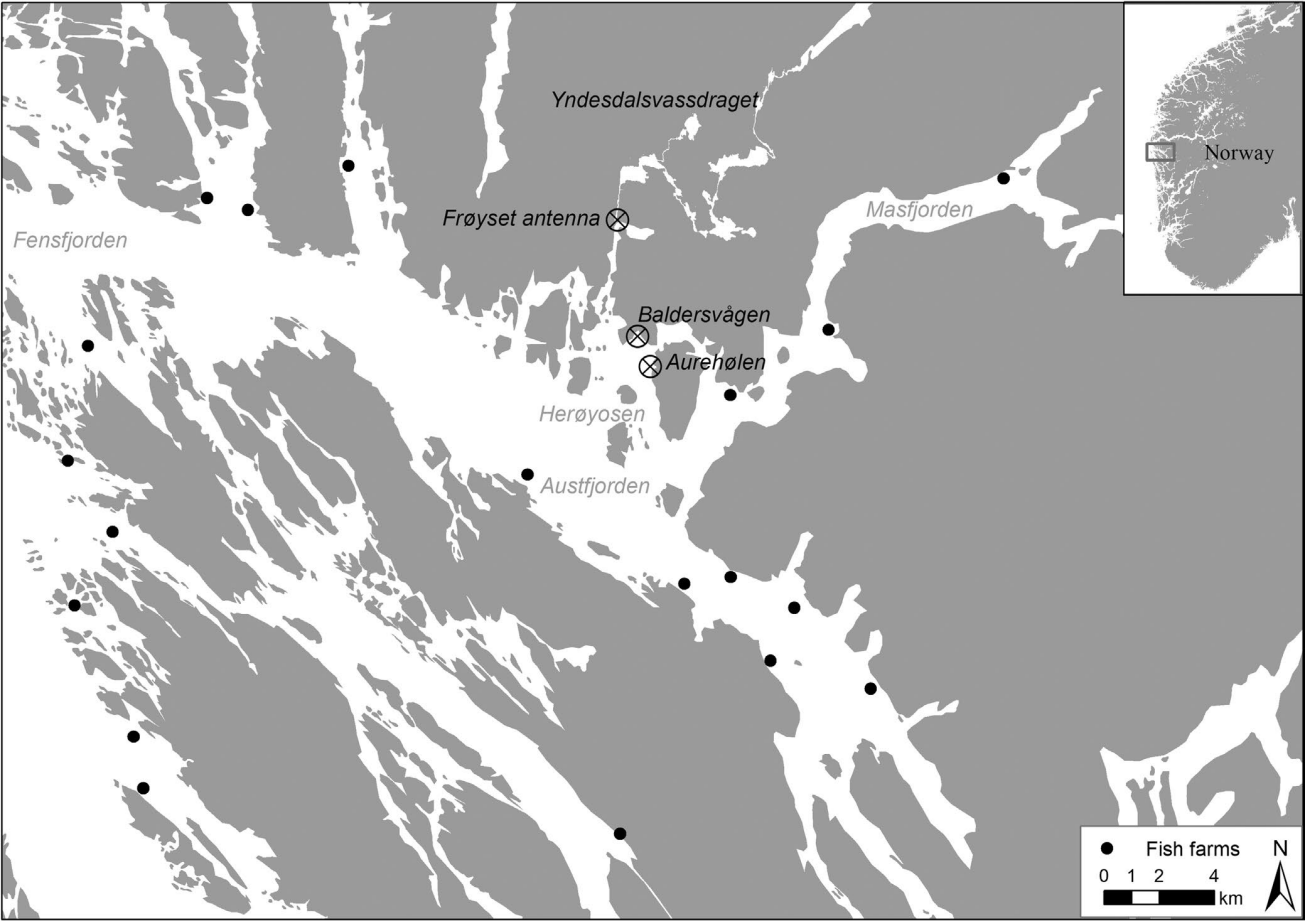
Map of area of capture (Baldersvågen and Aurehølen) and PIT antenna (Frøyset antenna at the inlet of the watercourse Yndesdal), and locations of active fish farms (solid dots). Name of local fjordnames are indicated. Note that the whole fjord system are called Fensfjord when talking about how many active farms were present in the system although there are more local names for smaller fjord sections (e.g., Austfjord and Masfjord).

Wild sea‐trout were captured early in the marine migration (springtime) and PIT‐tagged in the Herøyosen fjord basin in Vestland county, Norway, using trap‐nets at the locations Aurehølen and Baldersvågen (Figure 1). Herøyosen is located in an area where three fjords meet: Masfjorden in the east, Austfjorden in the south and west, and Fensfjorden in the north (Figure 1). Since 2015, this location has been one of the sentinel areas in Norway for sea lice densities, as part of the national salmon louse monitoring program (Bøhn et al. 2022). Nearly all trout in every year has been infested by salmon lice, with estimated median intensities exceeding the minimum threshold of 0.3 lice per gram fish (Table 1). Five kilometres north of Herøyosen the river Yndesdalsvassdraget enters the fjord in Frøyset (Figure 1). Yndesdalsvassdraget is the largest of the watercourses located in close proximity to the Herøyosen fjord basin. In total, there are > 15 aquaculture farm localities that are within the fjord system where the capture location is situated. The 15 closest localities (< 25 km sea distance) have a maximum permitted standing biomass of over 40,000 t of farmed fish within. In Norway the tolerable number of gravid female lice on farms are 0.5 lice per fish, except for a period during the spring (Week 16 to 21) where the tolerable levels are 0.2 lice per fish to reduce the likelihood of spill‐back effects on out‐migrating salmon smolts (Vormedal 2023). The two closest fish farms were approximately 6 and 5.5 km away from the capture location measured in sea distance. There was an alternating production cycle in the fish farms, where the closest farms were producing fish from 2018 to 2020, while other farms further away started production in 2019 (www.barentswatch.no). Consequently, in 2020, sea lice most likely originated from farms > 9 km away from the capture location, while in 2021 the lice originated from the closest farms. Output from a hydrodynamic model suggested that every year from 2020 to 2024 were patches of high aggregations of sea lice originating from fish farms within 6 km of the capture locations during spring and summer months (www.lakselice.no).

**TABLE 1:** Overview of the amount of salmon lice (prevalence, intensity, lpg) on sea trout in Herøyosen in the years 2020–2023. Not all of the fish were PIT tagged to minimise handling during days of high catches. Confidence intervals (CI) are bootstrapped confidence intervals using the boot package in R (Angelo and Ripley 2021).

Year	Week #	Prevalence %	Intensity [95% CI]	lpg [95% CI]	Range lpg	*N*
2020	21–26	99.6	53.2 [50.1–58.4.]	0.53 [0.49–0.58.]	0–3.30	488
2021	21–26	99.7	58.9 [52.0–68.6.]	0.64 [0.58–0.73.]	0–6.04	352
2022	21–26	93.7	73.8. [67.7–80.4.]	1.03 [0.92–1.15.]	0–7.36	523
2023	21–26	99.4	34.4 [33.7–37.9.]	0.52 [0.50–0.57.]	0–3.48	714

### 2.2 PIT‐System and Antennae Configuration

A PIT tracking system has been installed in Frøyset. The system consists of a half duplex (HDX) single antenna reader from Oregon RFID, which is connected via twinax cable to an automatic antenna tuner. The antenna body was made from 25 m of insulated, stranded earth wire (5.6 mm copper core diameter), which was then threaded into polyethylene plastic tubing (25 mm diameter). This was subsequently folded into a 12 × 0.6 m ladder‐like loop, anchored horizontally with rebar to the river substrate across the thalweg, and coupled to the antenna tuner inside a waterproof container. A marker PIT‐tag box was strapped onto the antenna to verify continuous operation (i.e., sentinel tag). The water level over the antenna may vary between ca 30 and 130 cm depending on river discharge and tide. The antenna's detection range was measured to 35 cm for 23 mm HDX PIT‐tags, while the range for 12 mm HDX tags was approximately 60% of the 23 mm HDX which corresponds to ~21 cm. Fish are often observed to swim relatively close to the bottom when entering rivers, thus a 35 cm range should give a high probability of tag detection (Pers. obs. G. Lehmann). The PIT‐system in Frøyset has been in continuous operation since May 4 2021. In addition, a hand held PIT reader was used to register any recapture of PIT tagged trout in the trap net.

### 2.3 Sampling Design

**FIGURE 2:**
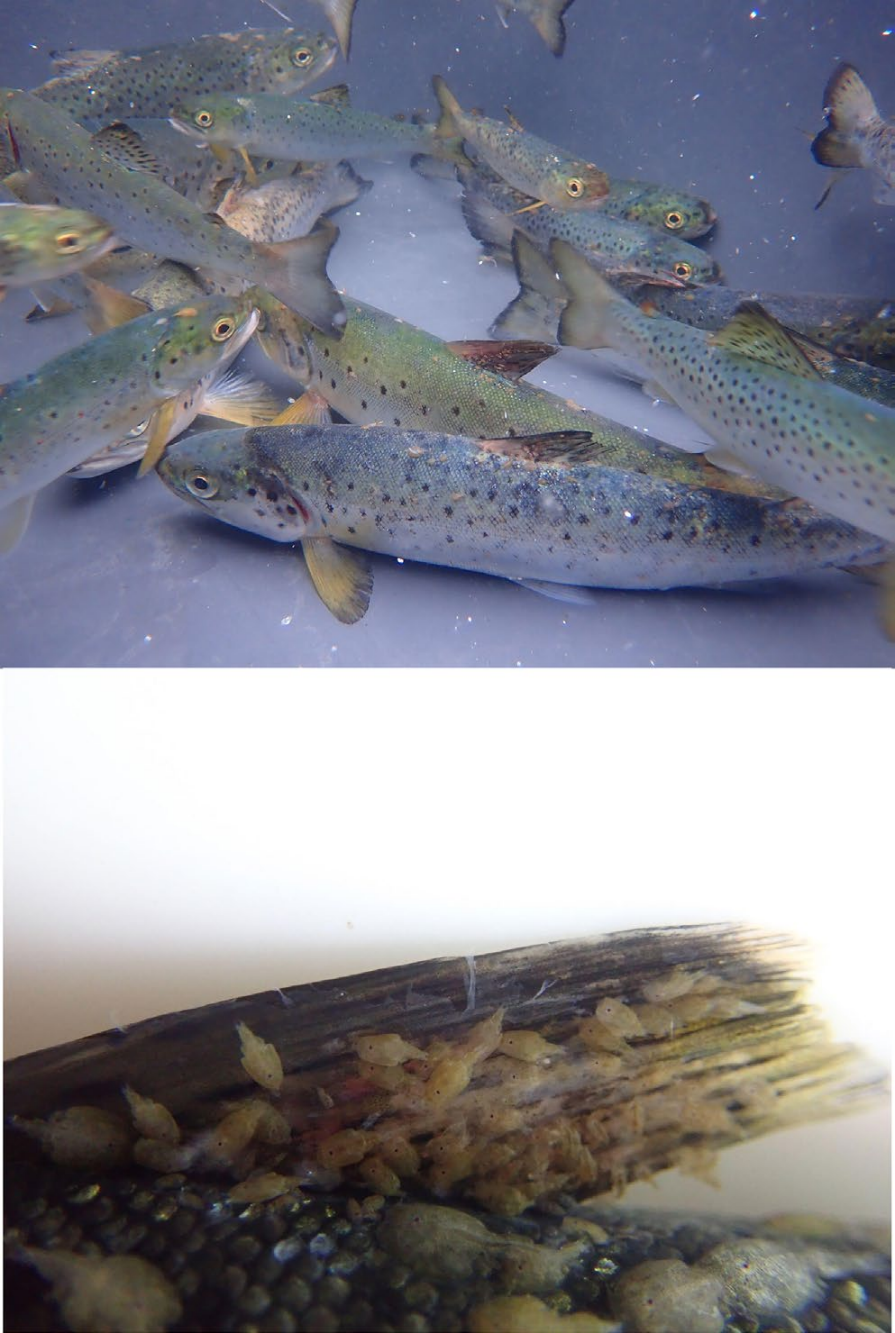
Pictures from capture of sea trout infested with salmon lice, where upper image is from holding tank and lower is a close up of the dorsal fin of a heavily infested sea trout during counting (Photos by Gunnar Lehmann and Bjørnar Skår).

Fish traps in Herøyosen were set up for 3–6 weeks within the period from May 20 to July 1, and were checked daily. The trap net's total length was 65–70 m and 5 m deep. It floats on the surface of the water and is anchored with seven or more 8–15 kg anchors which give the net height stability. The trap net consists of a lead net that is attached to the shore at a 90° angle, two wing nets, a central ‘heart’ department where the nets meet and fish enter the trap, and a tunnel that extends from the heart to a residence chamber. The trap net's construction and mode of operation is described in Barlaup et al. (2013). When caught, the fish were kept temporarily in 90 L tubs filled with seawater and brought ashore. All measurements were taken on live fish. Tricaine methanesulfonate (Finquel vet.) was used for anaesthesia at a concentration of 100 mg/L to sedate the fish so that lice could be counted without euthanizing them. Oxygen level and temperature in the anaesthetic tub were continuously monitored by a fixed instrument (limits: O_2_ saturation min 80%, Temperature max 17°C). Lice counts were performed in accordance with methods described in Bøhn et al. (2022) (counting all stationary and mobile stages, Figure 2). The fish was measured for length (mm) and weight (g), and scale samples and genetic samples were taken by removing a scale and taking a small adipose fin clip preserved on alcohol (data not shown). Finally, a scalpel (blade #15) was used to make a 2–3 mm longitudinal incision through the abdominal wall, followed by manual insertion of disinfected 12 mm (2020) or 23 mm (2021) HDX PIT‐tags into the abdominal cavity. The fish were then placed in a bath of fresh seawater to recover. Once they had recovered, they were released back into the sea from shore.

Water temperature in the fjord was measured using a Solinst LTC Logger positioned at a depth of 1 m inside the fish traps. During Weeks 21 to 26 in 2020, temperatures recorded at 12:00 ranged between 9.2°C and 19.4°C. For 9 days towards the end of the study period, temperatures were between 17.2°C and 19.4°C. In the corresponding period of 2021 (Weeks 21 to 26), temperatures ranged from 11.6°C to 17.1°C, with temperatures remaining below 17°C for all but 1 day during this period. Water supplied to the holding tanks for the fish, however, was pumped from a depth of 5 m. Consequently, its temperature was slightly lower than the water measured at 1 m depth in the traps. Temperature and oxygen saturation in the anaesthetic tank were continuously monitored using a WTW MultiLine Logger. This ensured that the investigations could be terminated if temperature or oxygen levels exceeded acceptable limits (17°C/85%).

### 2.4 Data Analysis

Trout tagged in 2020 and 2021 were subsequently detected on the PIT antenna in the river or they were recaptured in the trap and identified by handheld scanning of the PIT tag. Registrations from the same year were filtered out so that analysis was only carried out on fish that were redetected in future years. Two separate Cormack‐Jolly‐Seber (CJS) mark‐recapture analysis were constructed to estimate the survival to the subsequent year for the 2020 (detections from 2021 to 2023) and the 2021 cohort (detections from 2022 and 2023). The model also provided an estimate of detection probability (denoted as p in the CJS model). The model included time (i.e., recapture year) fish size, and lice (measured as lice per gram fish weight, lpg) as covariates to test the influence of these variables on survival probability (denoted as ɸ in the CJS model). In addition, we tested models with and without different detection probability of the different recapture years and whether having lice changed the probability of detection. Probability of detection (p) must be interpreted as a combination of the probability of the antennas registering a fish across all years, tag loss of individuals, and that a fish caught at the trap location was from a different river and was never again detected at any of the PIT readers (i.e., emigration from an open study system). Effects of lice on probability of detection had very little effect and for ease of presentation we have excluded this effect from the results.

The CJS model does not allow estimation of the detection efficiency of the last year, therefore the estimates of survival from the last year are excluded (i.e., survival from 2022 to 2023 and detection efficiency in 2023). The models were compared using the Akaike information criterion (AIC), and models with a delta < 2 were perceived equivalent (Burnham and Anderson 2002). All models was done in R version 4.2.1(R Core Team 2022).

## 3 Results

In 2020, 374 trout were captured and tagged, 30 of which were redetected in 2021 (8%), 10 of which were detected in 2022 (3%) and 6 of which were detected in 2023 (1.6%). Of the 10 detected in 2022 7 was also detected in 2021, while of the 6 detected in 2023, 5 of the fish were detected either in 2022 or 2023. At the time of tagging, 99.4% were infested by lice, and counts ranged from 0 to 324, with a median of 39 lice per fish. Lice per gram was 0–3.29. The size of the fish ranged from 21 to 68 cm with a median of 21 cm.

In 2021, 302 trout were captured, 32 of these were redetected in 2022 (11%) and 21 (7%) in 2023. Of the 21 detected in 2023, 19 were also detected in 2022. Among these, 97% were infested by lice with 0–607 lice per fish (median = 33.5) and lice per gram ranging from 0 to 2.68 (Figure 2). The length of the fish ranged from 21 to 68 cm with a median of 21 cm.

### 3.1 CJS Models

#### 3.1.1 Survival (ɸ)

For both the 2020 and the 2021 cohorts lpg (i.e., lice per gram fish weight) was a significant predictor of re‐detection. In both instances, there was equivalent support for an interaction between lice and year (deltaAIC < 2, Tables 2 and 3). In 2021, there were also three models with deltaAIC < 2 which also included the model without lice with only year as a predictor (deltAIC = 1.69, Table 3) indicating that the effect of salmon lice in 2021 was more uncertain. We chose to present the same simple model with only year and lpg as predictor for both years for consistency.

**TABLE 2:** AIC comparisons of CJS models of sea trout tagged in 2020 and registered in rivers the two following years, where npar is number of parameters in the model AIC is the Akaike information criterion, weight is the weight of the model and neg2lnl is the likelihood ratio.

Model	npar	AIC	DeltaAIC	weight	neg2lnl
ɸ(~time + lice)p(~time + lice)	8	315.7	0.0	0.37	299.7
ɸ(~time * lice)p(~1)	**7**	**317.1**	**1.4**	**0.19**	**303.1**
i(~time + lice + weight)p(~time + lice)	9	317.7	2.0	0.14	299.7
ɸ(~time + lice)p(~1)	5	318.5	2.8	0.09	308.5
ɸ(~time * l)p(~time + lice)	10	318.6	3.0	0.08	298.6
ɸ(~time + lice + weight)p(~1)	6	320.4	4.8	0.03	308.4
ɸ(~time * lice)p(~time)	9	320.4	4.8	0.03	302.4
ɸ(~time + lice)p(~time)	7	320.6	4.9	0.03	306.6
ɸ(~time + lice + weight)p(~time)	8	322.5	6.9	0.01	306.5
ɸ(~time)p(~1)	4	324.1	8.4	0.01	316.1

Bold indicate the selected model presented in table.

**TABLE 3:** AIC comparisons of CJS models of sea trout tagged in 2021 and registered in rivers the three following years, where npar is number of parameters in the model AIC is the Akaike information criterion, weight is the weight of the model and neg2lnl is the likelihood ratio.

Model	npar	AIC	DeltaAIC	weight	neg2lnl
**ɸ(~time + lice)p(~1)**	**4**	**273.3**	**0.0**	**0.20**	**265.3**
ɸ(~time + lice)p(~time + lice)	6	274.0	0.7	0.14	262.0
ɸ(~time * lice)p(~1)	5	274.7	1.3	0.10	264.7
ɸ(~time + lice)p(~time)	5	274.7	1.4	0.10	264.7
ɸ(~time)p(~1)	3	275.0	1.7	0.09	269.0
ɸ(~time * lice)p(~time + lice)	7	275.2	1.9	0.08	261.2
ɸ(~time + lice + mass)p(~1)	5	275.3	2.0	0.07	265.3
(~time + lice + mass)p(~time + lice)	7	275.5	2.1	0.07	261.5
ɸ(~time + lice + mass)p(~time)	6	276.1	2.7	0.05	264.1
ɸ(~time * lice)p(~time)	6	276.7	3.3	0.04	264.7

Bold indictate model presented in table.

Survival probability from the first to the second year, when number of salmon louse per fish weight, lpg, was 0 was 19.8% (CI 11.8%–31.3%) for the 2020 cohort and 17.3% (CI 10.6%–26.8%) for 2021. Survival probability from Year 2 to Year 3 was 46.5% (CI 26.5–67.6%) for the 2020 cohort when lpg was 0.

**FIGURE 3:**
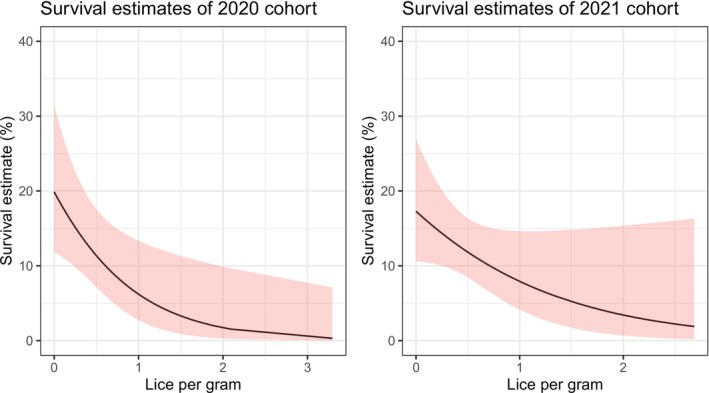
Survival probability from the CJS model fit for 2020 cohort and 2021 cohort, where the intercept is based on the survival the first year after tagging and the slope is from the effect of lice per gram fish mass (i.e. ɸ.lice). Red shaded area is 95% confidence intervals. The last year (ɸ.time2 and ɸ3.time3) for the two are not displayed because the uncertainty around the estimates is very large.

In 2020 survival probability (ɸ) after 1 year decreased to 14.2% when the lpg increased to 0.3 and 6% if lpg increased to 1 lice per gram (Figure 3, Table 4). This corresponds to an approximately ~73% decrease in survival for each unit of lice per gram. This further corresponds to a ~12% decrease in presumed survival when lpg is 0.1, 32% decrease when lpg is 0.3 and ~54% decrease when lpg is 0.6.

**TABLE 4:** Best‐supported CJS models by AIC for 2020, where se is standard error lcl is lower confidence interval and ucl is upper confidence interval. ɸ indicates survival probability as index of survival while p is detection probability, that is, the probability of being detected if the fish passes the antennae. ɸ.lice is the effect of lice per gram mass on survival probability, while p.lice is the effect of lice per gram mass on detection probability.

	Estimate	se	lcl	ucl
ɸ.(Intercept) (2021)	−1.39	0.31	−2	−0.78
ɸ.time2 (2022)	1.25	0.53	0.22	2.29
ɸ.time3 (2023)	2.15	0.91	0.37	3.92
ɸ.lice	−1.32	0.54	−2.39	−0.25
p.(Intercept)	0.5	0.48	−0.44	1.43

Similarly in 2021, survival probability (ɸ) after 1 year decreased to 13.7% when the lpg increased to 0.3 and 8% if lpg increased to 1 lice per gram (Figure 3, Table 5). This corresponds to an approximately ~58% decrease in presumed survival for each unit of lice per gram. This further corresponds to a ~8% decrease in presumed survival when lpg is 0.1, 23% decrease when lpg is 0.3 and ~41% decrease when lpg is 0.6.

**TABLE 5:** Best‐supported CJS models by AIC for 2021, where se is standard error lcl is lower confidence interval and ucl is upper confidence interval. ɸ indicates survival probability as index of survival while p is detection probability, that is, the probability of being detected if the fish passes the antennae. ɸ.lice is the effect of lice per gram mass on survival probability, while p.lice is the effect of lice per gram mass on detection probability.

	Estimate	se	lcl	ucl
ɸ.(Intercept) (2022)	−1.55	0.29	−2.12	−0.99
ɸ.time2 (2023)	2.31	0.46	1.4	3.21
ɸ.lice	−0.88	0.51	−1.88	0.12
p.(Intercept)	2.11	0.75	0.64	3.57

Neither for 2020 nor 2021 was mass a significant predictor of re‐detection. However, due to the calculations of lpg, mass and lpg are correlated and any additional effect of mass may be masked by the incliceion of lpg.

#### 3.1.2 Probability of Detection (*p*)

For both cohorts, the model with a constant detection probability (*p*) or both lice and time included as covariate was indistinguishable (deltaic < 2). For ease of interpretation, we chose to present the most parsimonious model with a constant p. Detection probability was 62.1% (CI 39.2%–80.7%) for 2020, and 90.1% (CI 68%–97.5%) for 2021 (Tables 4 and 5). Note that the more complex model changes the effect of lice on survival probability to a steeper relationship.

## 4 Discussion

Symbiotic relationships between parasites and hosts should lead to fitness benefits for the parasite at the expense of the host. Salmon lice is one of the most economically damaging marine parasites, affecting both farmed and wild fish populations (Costello 2009). In general, most research on lice‐salmonid relationships has focused on short‐term impacts of lice on their hosts (Bjørn and Finstad 1997) rather than the long‐term fitness ramifications of lice outbreaks in the wild (but see Vollset et al. 2014 and Krkošek et al. 2013, for example). Our study is among the first to estimate the relationship between long‐ term survival of sea‐run trout and salmon lice burden, revealing effects of lice parasitism on the lifetime fitness of sea trout, and show that the first year survival estimate decreases substantially with increasing infestation pressure.

The PIT tagging and mark‐recapture analysis provided clear evidence that lice infestations have carry‐over effects for sea trout, negatively affecting survival on subsequent years and thereby lifetime fitness. However, the estimated effect size with respect to inter‐annual survival (based on re‐detection) is less than what would have been estimated from laboratory studies using similar infection burden ratios (Bjørn and Finstad 1997). There are two major differences between laboratory and field trials when investigating how parasitism of salmon louse relates to survival; the first is that laboratory trials use experimental infections and randomised control treatment designs whereas field studies predominantly observe the number of lice on an animal with no manipulation other than tagging. Observational studies such as ours ignore underlying risk factors such as run timing and habitat selection that can affect exposure to lice. Second, and more important, fish in the wild can adjust their behaviour to account for lice parasitism (Halttunen et al. 2018; Mohn et al. 2020) whereas fish in laboratory trials do not have the same luxury. Impacted fish from our study may move back to freshwater earlier to encourage lice to detach and restore their osmotic balance, which should mitigate the impacts of lice on survival, although this will have trade‐offs such as reduced foraging time and therefore reduced growth and fecundity (aka fitness; Finstad et al. 2021). Survival was reduced by 70% and 52%, the two different years at a level of 1 louse per gram. This level of burden is substantially higher than what Taranger et al. (2015) has set as a lethal limit (0.3 lice per gram). It is important to note that this limit was defined as 100% mortality *or* that fish returned prematurely to freshwater. Prematurely returned trout are frequently observed during the month of June in rivers near the study site. In fact, a camera installed in the fish ladder of Yndedalsvassdraget has shown observations of lice infested sea trout returning to the river every year in June since 2021 (Gunnar Lehmann, unpublished data).

Although the study spans multiple years, the effect is not measured at the scale of the full trout life cycle. Lice parasitism affects the physiological functioning of trout and has also been documented to affect behaviour (Halttunen et al. 2018; Mohn et al. 2020) and life history (Hedger et al. 2021). For example, Vollset et al. (2014) observed that lice parasitism explained part of the variance in sea age at maturity for Atlantic salmon, meaning that growth was compromised among parasitized individuals and lifetime fitness was also affected by the parasite. Whereas Atlantic salmon mostly spawn only one time, with < 10% spawning multiple times (Birnie‐Gauvin et al. 2023), trout are much more frequently repeat spawners and are expected to have a longer lifespan than salmon (Klemetsen et al. 2003). Tracking sea‐run trout from Herøyosen using PIT tags across multiple years provided an inference of a lifetime fitness impact of lice infestation, which would not be possible with acoustic transmitters, which are limited by battery life, or with mark‐recapture using external tags, which depend on manual recapture. PIT telemetry was therefore ideal for this study. Although we could not account for potential tag loss or imperfect detection by the antenna, these are not expected to differ for trout with high or low lice burden and therefore these limitations should not affect our main findings.

Our study does not consider population impacts on size at maturation, a key component of lifetime fitness, which are highly likely to be impacted by the altered behaviour of sea trout. The marine migration of trout is believed to be a strategy employed to exploit productivity of the ocean during spring and summer months for more rapid growth than is possible in the more oligotrophic freshwater rivers of northern Europe such as those in Yndedalsvassdraget (Klemetsen et al. 2003; Birnie‐Gauvin et al. 2023). Indeed, sea trout should have faster growth and higher fecundity than resident trout (Jonsson and Jonsson 2006). If the marine migration is cut short, however, feeding will be insufficient to reap the full benefits of migrating to sea and the physiological costs of managing infection may reduce overall fecundity (Finstad et al. 2021). Premature return of infested sea trout to rivers may be a strategy to limit the impacts of lice on the individual but does not completely offset the damages incurred from the parasitism and it is unlikely that trout can compensate for the losses to their growth. Consequently, estimating the decreased survival due to salmon lice does not account for alterations to growth and fecundity and therefore our estimates should be viewed as an estimate of lice parasitism based on minimal covariates.

Although these mark‐detect data are unique in the context of sea‐run trout exposure to parasites, it is important to consider that the relationship may be location and context dependent, and our results should not be extrapolated too broadly to populations with different migratory behaviours. Sea trout are highly plastic and different populations will have different life history and behaviour. For example, trout may adjust their marine residence time to account for conditions in the home river, with fish originating from small systems migrating back to the estuary or ocean after spawning to overwinter in saline water rather than in the river (Limburg et al. 2001). Some trout populations may remain close to the home river to capitalise on feeding conditions in productive estuaries whereas fish from other populations will move long distances away from the home river to seek out better feeding opportunities (Lennox et al. 2024). Indeed, we would expect trout originating from larger rivers with higher rates of freshwater discharge to the estuary would have different risk factors and behaviours in response to parasite burden. Replicated work using passive tagging at a broader landscape scale will help to understand how marine habitat availability interacts with fish size and parasite burden to generate a more holistic concept of the fitness consequences of lice burden on sea‐run trout.

There are other factors known to affect the return timing and survival of sea trout in the ocean that warrant further consideration. Marine migration timing is closely related to fish size and condition, with significant variation across watersheds due to differences in hydrology and feeding/growth conditions (Lennox et al. 2024). These factors can modulate an individual's encounters with food, predators, parasites, and anglers, influencing overall survival rates (e.g., Kristensen et al. 2019; Eldøy et al. 2021; Jensen et al. 2022). For example, larger fish are often subject to size‐dependent mortality risks during their marine phase, with survival probability being directly influenced by size (Paterson et al. 2021). Additionally, microbial interactions, such as those with bacteria and viruses, can alter habitat use, thereby affecting exposure to sea lice (Lennox et al. 2020). While our study emphasises the impact of lice burden on survival, it is important to acknowledge that these other factors also play roles in the survival and return timing of sea trout. In the context of our study, it is important to clarify the distinction between survival and redetection. Sea trout exhibit highly flexible life history patterns, with some individuals continuing to migrate annually after their first marine migration, while others may opt to remain in freshwater for the remainder of their lives. This variation in migratory behaviour is not only individual‐specific but also watercourse‐specific, as observed in various Norwegian rivers (Paterson et al. 2021). Consequently, the absence of detection by the antenna does not necessarily indicate mortality; rather, it may reflect a decision by the fish to forgo further marine migrations. This distinction is crucial to accurately interpret our findings, because it underscores that non‐detection could be due to behavioural choices rather than a direct consequence of mortality. As a result, our estimated effect of salmon lice on survival may be overestimated if non‐detection is interpreted as mortality when the fish has actually survived but chosen not to migrate. Conversely, it could underestimate the impact if lice burdens are causing a behavioural shift that leads to higher probability of being detected by the antennae in the lower part of the river, thereby obscuring the true fitness costs associated with lice parasitism.

## 5 Conclusion

Sea‐run brown trout are one of the most important anadromous species connecting inland and marine ecosystems in the north Atlantic. However, there is increasing recognition that trout populations are extremely vulnerable to the impacts of sea lice, with evidence for alterations in marine living area, marine living time (Finstad et al. 2021), and, here, survival. We revealed a relationship between lice burden and survival of trout across multiple years that demonstrated how louse infestations affect estimated survival across years. This finding supports results from laboratory studies that show the impacts of lice on trout physiology and survival (Bjørn and Finstad 1997; Birkeland 1996), but add in the field contexts where trout has the option to seek out sources of freshwater. PIT tag systems provide the ideal methodology for studying multi‐year impacts of parasitism on trout because the passive tags can be used for small fish with minimal tag burden and the tag does not have a battery that will expire. Therefore, additional effort can be allocated towards generating a better understanding of how lice dynamics influence sea‐run trout at a more landscape scale via replicated research using PIT systems and lice counts across scales. Future research is clearly needed to estimate the fitness costs of lice parasitism and determine how the rapid loss of trout may affect coastal and riparian cycling at a landscape scale.

## Author Contributions

**Rosa Maria Serra‐Llinares:** conceptualization (equal), methodology (equal), resources (lead), writing – original draft (equal), writing – review and editing (equal). **Robert J. Lennox:** methodology (equal), supervision (equal), validation (equal), writing – original draft (equal), writing – review and editing (equal). **Knut Wiik Vollset:** conceptualization (lead), data curation (equal), formal analysis (lead), funding acquisition (supporting), investigation (supporting), methodology (lead), project administration (supporting), resources (lead), software (lead), supervision (equal), validation (equal), visualization (lead), writing – original draft (lead), writing – review and editing (lead). **Gunnar Bekke Lehmann:** conceptualization (lead), data curation (equal), funding acquisition (lead), investigation (equal), methodology (equal), project administration (equal), supervision (equal), writing – original draft (equal), writing – review and editing (equal). **Bjørnar Skår:** conceptualization (equal), data curation (equal), investigation (lead), visualization (equal).

## Conflicts of Interest

The authors declare no conflicts of interest.

## Data Availability

All data used in the analysis is available at 10.5281/zenodo.14382696
